# Promoting sensitive parenting in ‘at-risk’ mothers and fathers: A UK outcome study of Mellow Babies, a group-based early intervention program for parents and their babies

**DOI:** 10.1371/journal.pone.0245226

**Published:** 2021-02-03

**Authors:** Aigli Raouna, Ruaridh Malcolm, Raquib Ibrahim, Angus MacBeth

**Affiliations:** 1 Mellow Parenting, Glasgow, United Kingdom; 2 Department of Clinical Psychology, School of Health in Social Science, The University of Edinburgh, Edinburgh, United Kingdom; University of Lleida, SPAIN

## Abstract

**Background:**

The objective of this study was to evaluate the effectiveness of Mellow Babies (MB) in the UK. MB is a 14-week early parenting intervention program that is delivered in groups and is targeted at ‘at-risk’ parents (both mothers and fathers) and their babies up to 18 months old.

**Method:**

The study used a pragmatic pre-post intervention design. Outcomes were parental mental health, parenting confidence, quality of life, socio-emotional development of children, and perceived parent-child relationship. Fifteen groups representing *n* = 91 parent-baby dyads were recruited across the UK between 2017–2018. The sample consisted of 10 Mellow Mums groups (70 mother-baby dyads) and 5 Mellow Dads groups (21 father-baby dyads). Intention-to-treat and ‘completer’ analyses were performed.

**Results:**

Findings suggest short-term positive outcomes for parents attending MB. Completion of the program was associated with significant improvements in anxiety and overall wellbeing, parenting confidence, and perceived closeness of the parent-child relationship. The significance of these improvements, except for parenting confidence, was maintained in the intention-to-treat analysis. MB engaged and retained a high proportion of parents who could be considered ‘at-risk’ and benefitted fathers and mothers attending the intervention equally.

**Conclusions:**

This is the first prospective study to explore MB participation for both mothers and fathers and to indicate engagement and potential benefits specifically for ‘at-risk’ parents. Findings further demonstrate the effectiveness of MB as an early intervention program for parents experiencing psychosocial difficulties. Replication by studies using a contrast or control group also incorporating follow-up data would further improve the evidence base for MB.

## Introduction

The early years of life present a unique window of opportunity to lay foundations for healthy lifespan development [1]. Research on early childhood highlights the importance of the quality of early social interactions between infants and primary caregivers on children’s immediate and long-term socio-emotional, cognitive, and behavioral development; mental health; and academic competence [2, 3]. Indicators of low family economic and psychosocial resources, such as poor parental mental health [4, 5], lack of social support, and multiple deprivation indices such as housing and financial worries, unemployment, substance use, low parental education, and single parenthood [6–8] increase the risk for suboptimal early development in children. These contextual risk factors tend to co-occur, increasing family stress, and limiting the practical and emotional support parents can provide their children [9–11]. Consequently, there are significant potential public health benefits for targeted delivery of early intervention programs with parents experiencing one or more of the above risk indicators (‘at-risk’ parents).

### Early parenting interventions for ‘at-risk’ parents

Compared to targeted programs, universal parenting programs have lower effectiveness and are less likely to enable ‘at-risk’ parents to participate in beneficial support services [12–14]. Enrolment, attendance, and engagement of ‘at-risk’ parents, and especially of fathers, pose particular challenges to program delivery. Multiple factors may constitute barriers, including personal life factors (e.g. mental health, lifestyles, and limited resources) and program-specific factors (e.g. delivery, gender-specific content, and support arrangements) [15–17]. Furthermore, despite emerging evidence illustrating the significance of the father-infant interaction on child developmental outcomes, co-parenting remains undervalued as contrasted with mothering, mirrored in the design of programs, their delivery, and their evaluation [15, 18, 19].

The majority of current evidence-based parenting programs in the UK, such as Incredible Years [20] and Triple P [21], primarily target families with children over 2 years and tend to focus on increasing parental perceived competence, on management of children’s behavior and/or on parent-child interaction; without a corresponding emphasis on parental mental health. Existing research highlights that if psychosocial risk factors (e.g. psychiatric disorder, family violence, and substance misuse) are unaddressed, improvements in parenting capacity and parent functioning are likely to be minimal [22, 23]. On the other hand, treating parental mental health symptoms alone does not significantly improve parent-child interaction quality and parenting competence [24, 25]. Therefore, for parents with multiple and complex needs, a balanced approach including addressing underlying issues and building parenting capacity is essential [26].

### Mellow Babies: An early parenting intervention

Mellow Babies (MB) is part of the Mellow Parenting (MP) family of early intervention programs (https://www.mellowparenting.org/), specifically targeting parents who experience psychosocial difficulties, with children up to 18 months of age. The intervention is free, group-based, and includes gender-specific postnatal programs (Mellow Mums and Mellow Dads) that run separately for 14 weeks–one full day a week–allowing parents to strengthen their social support [27]. Parallel to parent groups, childcare groups are also offered for free for participants’ young children. Mechanisms of change are via developing parental sensitivity and attunement, in line with attachment-informed interventions [28]. The interventions also incorporate parental mental health strategies (e.g. cognitive-behavioral strategies for ameliorating parental depression and anxiety), parent-child relationship components (e.g. joint lunchtime activities), and “homework” (i.e. new skills to try out at home). Use of strength-based video feedback and interactive tasks are key to program delivery, consistent with best practice in evidence-based parenting [10, 28].

Mellow Babies employs strategies to enhance engagement of ‘at-risk’ parent-infant dyads by providing transport, childcare, and meals, and by using free or inexpensive materials for parent-child activities, with parents encouraged to “have a go” at home. Parent-baby dyads who attend at least 70% of the group sessions (i.e. at least 10/14 sessions) are considered program completers. Non-specialists with professional experience in the field can deliver MB- following training. Ongoing supervision for practitioners is provided, which is essential for their accreditation. Two to three practitioners facilitate the group, of which at least one matches the gender of the parents in the group.

Mellow Parenting has a growing evidence base demonstrating medium-size effects for mothers’ mental health, parenting skills, and child outcomes [29–31]. These are also supported by qualitative studies [32–34]. However, there has been little evidence specific to the Mellow Babies intervention, or the effectiveness of MB with fathers. Specifically, the most recent MP outcome study exploring routine data collected by group facilitators [29] reports findings from a combined sample of mothers attending either a Mellow Babies or a Mellow Toddlers group. Additionally, the two studies identified in a recent MP meta-analysis [30] as having a specific focus on MB had a very small sample of mothers (both n<20), and one of the intervention designers was directly or indirectly involved (the nature of the involvement is unclear), thus reporting on the efficacy (i.e. the performance of an intervention under ideal and controlled circumstances) rather than the effectiveness (its performance under 'real-world' conditions) of MB.

### Aims & hypotheses

The objective of the current study was to evaluate the effectiveness of MB using a pragmatic, prospective implementation trial, addressing the aforementioned methodological limitations of previous studies. We aimed to mitigate previously reported bias and limitations [30] through publication of the trial protocol as a registered report (performance bias), by accounting for dropout participants in our results (attrition bias), collecting data independently (not by group facilitators) and ensuring intervention fidelity in the participating multi-site and naturally occurred groups (detection bias), and reporting conflicts of interest (reporting bias).

We intended to explore the effectiveness of MB, focusing both on the individual as well as the relational and child-related changes. Specifically, we evaluated psychological, parental, and behavioral changes in parents, changes in the perceived relationship quality with their infants, and the socio-emotional development of their infants. This is the first evaluation of MB with a specific focus on engaging ‘at-risk’ parents and the first to explore the effectiveness of MB with father-infant dyads.

Our specific research questions were:

1. Is attending MB associated with improvements in parental mental wellbeing, parenting confidence, and quality of life?
2. Do fathers engage with the MB program and, if so, are there differences in outcomes compared to mothers?
3. Do ‘at-risk’ parents engage with the MB program and, if so, are there any differences compared to not ‘at-risk’ parents?
4. Is participation in MB associated with improvements in children’s early social and emotional development?
5. Is participation in MB associated with improved parent-infant relationships?

We hypothesized that attendance at an MB group would be associated with improved mental wellbeing, parenting confidence, and increased life satisfaction. Further, we hypothesized that participating fathers would engage and benefit to the same extent as mothers due to the gender-specific delivery of programs tailored to parents’ unique needs both directly and indirectly in terms of content, modelling of male and female group facilitators accordingly, and access to peer support. Similarly, we hypothesized that ‘at-risk’ parents would engage and benefit to the same extent as not ‘at-risk’ parents due to the engagement strategies employed by MP such as provision of transportation, meals, and parallel childcare that would facilitate parents’ access and commitment to the program, alleviating practical issues that would otherwise function as barriers. Furthermore, we hypothesized that children’s early social and emotional development would be improved after attending the MB program; and that parents would report improvements in their relationship with their infant.

## Methods

### Ethical approval

Ethical approval for this study was granted by the Research Ethics Committee of the School of Health in Social Science, University of Edinburgh. The study was registered with ISRCTN: Registration number ISRCTN17621046.

### Design

This study used a pragmatic pre-post intervention design. We used a pragmatic trial design as this method allowed us to evaluate the effectiveness of MB in ‘real-life’ routine practice conditions, achieving maximum external validity and producing results that can be generalized and applied in UK routine practice settings [35].

Despite multiple efforts to recruit a control group (as per protocol), practical challenges (such as tight timeframe, limited resources, and resistance from group facilitators to use their activities as a comparison to MB intervention) resulted in the recruitment of only seven parent-infant control dyads. As there was insufficient power to conduct meaningful analyses, we opted to remove the control group and proceed with within-and-between subjects’ analyses.

### Identification of MB groups

Identification of MB groups used opportunistic sampling, with participants invited via MB groups starting in the UK between February 2017 and September 2018. An invitation poster for the study was sent to all MP trained practitioners in the UK via email and was shared on the MP website and social media pages. To ensure program fidelity, groups were only eligible if at least one of the group practitioners had previous supervised experience in delivering MB. Additionally, MB groups had to be able to implement all elements and values of MB to be eligible for this research project (e.g. at least one gender-specific practitioner in the group, weekly sessions including strength-based video feedback, free parallel childcare, parent-infant joint lunchtime activities, and provision of transportation and meals). Fifteen groups were recruited, of which 10 were delivered in Scotland (5 Mellow Mums and 5 Mellow Dads), 3 in England (Mellow Mums), and 2 in Northern Ireland (Mellow Mums). Each group received an incentive of £250 towards the cost of a group activity of their preference (e.g. zoo visit).

### Recruitment of parents in MB groups

Participating services facilitated the recruitment of parents via MP referral pathways (i.e. referred by a health-related professional such as a health visitor, or, less often, self-referred). Practitioners discussed with referred parents in order to assess their eligibility to participate in an MB group: All parents had to have at least one child under the age of 18 months that could attend at lunchtime and in joint activities each week, and parents had to agree to have a video recorded during a caretaking activity (e.g. during feeding). Although MP does not advise screening for specific issues, parents referred to the groups are often identified as experiencing a mental health issue (commonly depression and/or anxiety), isolation, unemployment, domestic violence, drug or alcohol misuse, difficulties in parental role, social work involvement, and involvement in child protection services.

Participation in the research project was voluntary and confidential, without affecting parents’ participation in the MB group. Parents were informed of the research project in their initial contact with the group facilitators and, either before or during the first group session, two members of the research team visited each group to provide face-to-face information and answer any questions regarding the study. All participants who agreed to take part in the project signed a consent form and entered a prize draw to win one of six £40 supermarket vouchers.

### Procedure

The research team consisted of three experienced MSc-graduate researchers employed by MP who were not blind to the study aims (AR, RM, RI). An independent researcher supervised the procedure, analysis, and interpretation of the study findings, thus mitigating risk of bias (AM). Researchers collected the data during a 1:1 session with each parent, in a private space within the group service facilities at two-time points (T): T1 –pre-group (baseline) and T2 –post-group. Data collection for T1 lasted approximately 30 minutes per participant and was organized after the information and consent meeting. Data collection for T2 was scheduled between weeks 12 and 14 of the program and each session lasted approximately 45 minutes. Program fidelity across the sites was ensured at the inclusion point of this project and was monitored during the program via reflective consultation of practitioners with senior MP trainers.

### Measures

#### Demographics

At baseline, participants were asked their age, nationality, mental health history, postcode (as a proxy for socioeconomic status), referral source, as well as marital, educational, and employment status alongside their children’s age, gender, residence, and contact status.

The following questionnaires were completed both at T1 and T2. Parent-infant video observation recordings were also collected at T1 and T2. At T2, semi-structured interviews were conducted with participants, group facilitators, and child-care workers, however, the current study focuses on the quantitative outcomes of the project and therefore only the quantitative measures will be reported in detail.

#### Psychological distress

The Brief Symptom Inventory-18 (BSI-18) [36] is an 18-item self-report measure of psychological distress. Items can be scored from "Not at all = 0” to "Extremely = 4" and raw scores are converted to t-scores based on gender-specific normative data from a non-clinical population. BSI-18 includes three subscales that assess individual symptom constellations (Depression, Anxiety, and Somatization) and one overall subscale that captures the intensity of global psychological distress (Global Severity Index) during the last two weeks. The reliability and validity of the questionnaire have been demonstrated in several studies for both community and clinical populations [37–39]. For the current sample, reliability at T1 was *α* = .90 for the overall scale and *a* = .80, .83, and .74 for subscales. Respectively, at T2 reliability was *α* = .93, .87, .87, and .80.

#### Parenting confidence

The Karitane Parenting Confidence Scale (KPCS) [40] is a 15-item self-reported instrument designed to measure the perceived self-efficacy of parents with children aged 0–12 months. KPCS scores range from 0 to 45, with higher scores representing higher parenting confidence. Scores can be assigned into four categories: ‘non-clinical’ (40 or more), ‘mildly clinical’ (36–39), ‘moderately clinical’ (31–35), and ‘severely clinical’ (31 or less). The cut-off for clinically low parenting confidence is <39. The KPCS has good internal consistency, test-retest reliability, and construct validity, including association with stress and depression constructs [40, 41]. Reliability at T1 was *α* = .58 and at T2 *α* = .53 in the current sample.

#### Quality of life, enjoyment and satisfaction

The Quality of Life, Enjoyment and Satisfaction Questionnaire—Short form (Q-LES-Q-SF) [42, 43] is a 14-item self-report instrument including items on daily functioning such as work, physical health, social relationships, family relationships, ability to function in daily life, and overall well-being. The raw total score ranges from 14 to 70, which can be transformed into a percentage of the maximum possible score, with lower scores indicating poorer quality of life enjoyment and satisfaction. The Q-LES-Q-SF has good to excellent internal consistency, test-retest reliability and construct, criterion, and convergent validity [44–46]. We report an alpha of *α* = .85 at T1 and *α* = .88 at T2.

#### Child socio-emotional development

The Ages and Stages Questionnaire: Social-Emotional, Second Edition (ASQ:SE-2) [47] is a parent-completed, developmentally specific questionnaire that focuses on young children’s social and emotional development (i.e. self-regulation, compliance, social-communication, adaptive functioning, autonomy, affect, and interaction with people). This study used the 2 (T1: *α =* .58), 6 (T1: *α =* .56, T2: *α =* .52), 12 (T1: *α =* .77, T2: *α =* .53), 18 (T1: *α =* .91, T2: *α =* .86) and 24-month old (T2: *α =* .89) questionnaires, with 15, 24, 29, 33 and 27 items respectively. The total score of each questionnaire indicates whether the social-emotional development of the child appears to be below cut-off (1: “on schedule”), close to cut-off (2: “review behaviors of concern and monitor”) or above cut-off (3: “further assessment with a professional may be needed”; 4: score is at the “90^th^ percentile” above cut-off, indicating the most ‘at-risk’ cases). Psychometric studies based on normative samples show high internal consistency, test-retest reliability, sensitivity, and specificity of the measurement [47].

#### Parent-child relationship

The “Tunnel” is a non-standardized visual analog scale developed by the MP Evaluation team to capture participants’ perceived closeness to their child throughout the program. An additional mid-group time point (TM) was collected by group practitioners for this study. The “Tunnel” is a respondent-friendly subjective task in which parents are encouraged to put a cross on a 10cm line, based on how they feel their relationship with their child is at the moment. The right edge of the line (point 10) represents an ideal relationship, while the left edge (point 0) represents a relationship that is far away from their ideal.

#### Feedback questionnaire

During T2 parents completed a non-standardized, 10-item, 5-Likert scale (from 1: Strongly Disagree to 5: Strongly Agree) feedback questionnaire about their experiences in the MB group and their intentions to further engage with the service that delivered the MB group. Questions explored whether parents found the group helpful for themselves, their babies, and their relationship, whether they would like to keep in touch with the service and the other members of the group. and whether they feel more confident to ask for help should they need it after completing the MB program. Reliability was *α* = .85 in the current sample.

#### Attendance and involvement with child protective services

After group completion, practitioners provided information about parent’s attendance and changes in their involvement with child protection services. Changes in child protection involvement were recorded from before to after the MB program, with the options: No Involvement, Step Up, Step Down, or Involvement Stayed the Same. Family involvement with child protection services was considered an important factor to monitor as it may act as a proxy indicator for significant deterioration or improvement in the quality of the parent-child relationship, as observed by an independent specialist body.

### Data analysis

Differences between program completers and non-completers were assessed using independent t-tests. Parents were categorized as either ‘at-risk’ or not ‘at-risk’. A parent was ‘at-risk’ if they lived in SIMD (Scottish Index of Multiple Deprivation: http://simd.scot/2016/#/simd2016/BTTTFTT/9/-4.0000/55.9000/) decile 1 or 2 (for Scotland-based families only; SIMD data can be split into deciles with decile 1 representing the 10% most deprived postcodes and decile 10 representing the 10% least deprived postcodes); if they reported experiencing a mental health issue; or if they reported being a single parent and being unemployed. A chi-square analysis was then utilized. Differences between baseline scores of mothers and fathers were compared using independent t-tests. To assess change in parents from before to after the group intervention (MB completers), paired t-tests and Wilcoxon signed-rank tests were used where appropriate. An intention-to-treat analysis using multiple imputation (MI) was also carried out to account for missing data at T2 from MB non-completers, improving the validity of our findings. Multiple imputation was preferred over single or mean derived imputation for missing values because of its strength to incorporate the variance of estimations derived across multiple datasets, providing unbiased standard errors and a robust method for dealing with a high rate of missing data that has been successfully applied in psychotherapy outcome trials [48]. All statistical analyses were conducted using SPSS v24.0. All tests were two-tailed with a significance level of *p* = 0.05 unless otherwise stated. Effect sizes were reported using Cohen’s *d*.

## Results

### Participant flow and baseline characteristics

**Fig 1:**
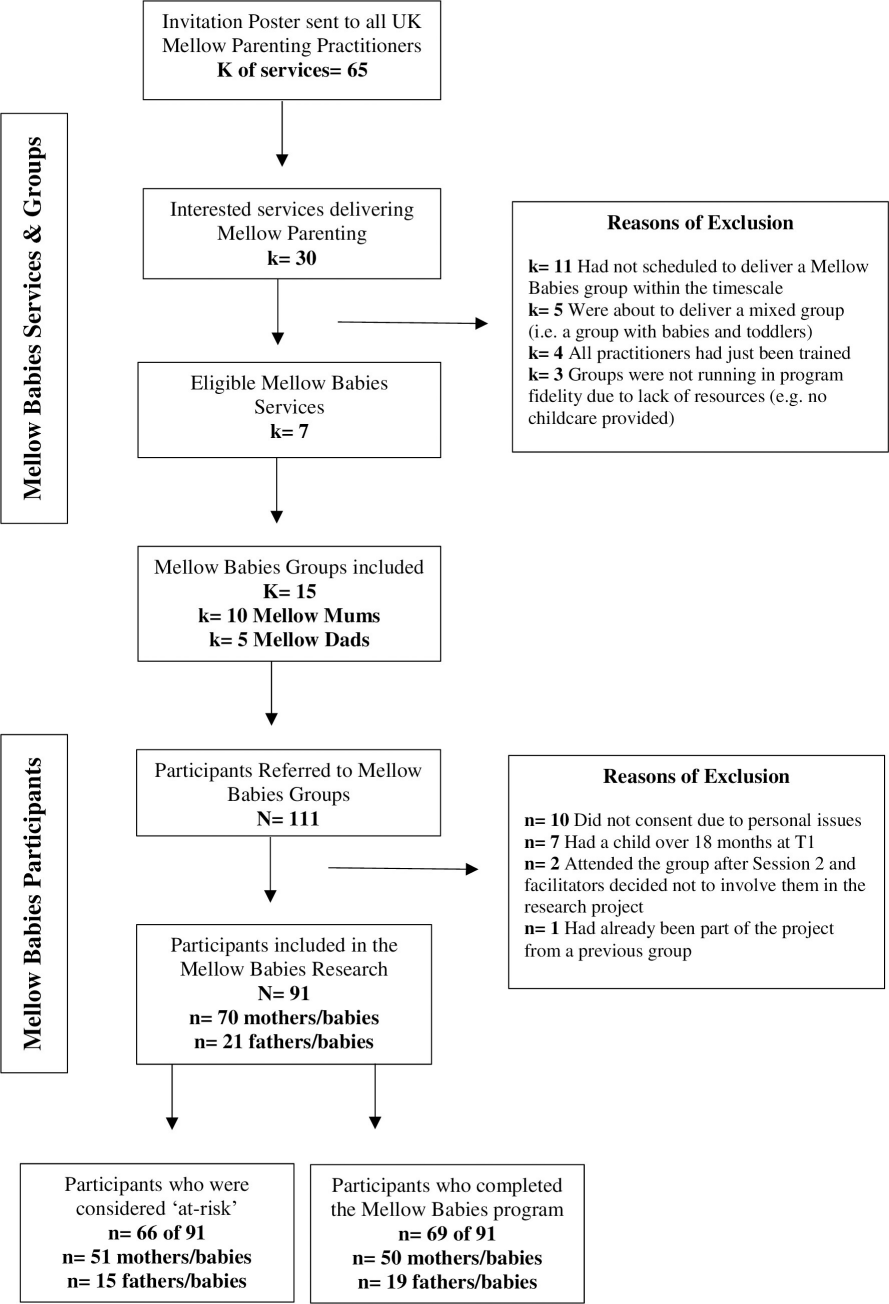
Mellow Babies research project: Services, groups and participants flow chart.

As illustrated in Fig 1, of the 111 parent-infant dyads recruited in the participating MB groups, 91 consented to take part in the research (70 mother-infant and 21 father-infant dyads). Twenty-two dyads did not complete the program, giving an attrition rate of 24.18% (0–44% range amongst groups). Of non-completers, 9% (2) were fathers. Those who completed the program attended an average of 80% of the 14 MB sessions.

Table 1 displays the baseline demographics of this study. The mean age for mothers was 24.91 (*SD* = 6.04) years, for fathers 28.04 (*SD* = 8.65) years, and for children 8.5 (*SD* = 5.19) months. The majority of the parents self-identified as British, unemployed, and receiving state benefits. More than one-third of participants were single parents and 19.78% of the children in the group did not live with the parent attending the group. Approximately half of the participants reported experiencing mental health issues, with the most common being anxiety and depression. The majority of parents were referred by Family Support Workers (26 mothers: 37.14%, 10 fathers: 47.62%), Social Workers (10 mothers: 14.28%, 7 fathers: 33.34%), and Health Visitors (25 mothers: 35.71%).

**Table 1:** Baseline demographics.

Study Variable		Mothers	Fathers	
**Age in years *(SD)***		24.91 (*6*.*04*)	28.04 (*8*.*65)*	
**Nationality [*n* (%)]**		***n* = 70**	***n* = 21**	
British		63 (90)	19 (90.48)	
Other		7 (10)	2 (9.52)	
**Education [*n* (%)]**		***n* = 70**	***n* = 21**	
Did not finish school		8 (11.43)	3 (14.29)	
Still at school		1 (1.43)	1 (4.76)	
Secondary school		24 (34.28)	11 (52.38)	
Vocational education		29 (41.43)	5 (23.81)	
Further education		8 (11.43)	1 (4.76)	
**Employment [*n* (%)]**		*n* = 69*	***n* = 21**	
Full-time employment		11 (15.94)	3 (14.29)	
Part-time employment		6 (8.70)	0 (0)	
Unemployed–with benefits		38 (55.07)	15 (71.42)	
Unemployed–no benefits		14 (20.29)	3 (14.29)	
**Relationship status [*n* (%)]**		***n* = 70**	***n* = 21**	
Single		28 (40.00)	6 (28.57)	
In a relationship, not co-habiting		7 (10.00)	4 (19.05)	
Co-habiting		22 (31.43)	7 (33.33)	
Married		13 (18.57)	4 (19.05)	
**History of mental health conditions [*n* (%)]**		*n* = 64*	***n* = 21**	
None		29 (45.31)	12 (57.14)	
Depression		9 (14.06)	1 (4.76)	
Anxiety		11 (17.20)	0 (0)	
Depression & Anxiety		13 (20.31)	4 (19.05)	
Other		2 (3.12)	4 (19.05)	
**Children in group (*n* = 91)**				
**Age in months (SD)**	8.5 (5.19)	**Residential status [n (%)]**		
**Gender [n (%)]**		Lives with parent		73 (80.22)
Female	44 (48.35)	Lives with other family member		9 (9.89)
Male	47 (61.64)	Supervised contact		9 (9.89)

* Discrepancies in *n* are due to mothers’ decision to not disclose the specific information.

#### 'At-risk’ parents

Based on our criteria, 73% (66) of our sample (91) were considered ‘at-risk’, of which 72% (51) were mothers. Looking at SIMD data, 23% (14) of our Scottish sample (63) were in SIMD decile 1 and 18% (11) were in SIMD decile 2. A chi-square goodness-of-fit analysis suggested statistically significant over-representation of low SIMD in the sample, compared to expected values if postcodes in our sample were distributed evenly as in the wider population (*χ*^*2*^(8) = 30.90, *p* < 0.001). ‘At-risk’ parents reported higher scores at the ‘Tunnel’ task (*M* = 6.61, *SD* = 2.83) compared to not ‘at-risk’ parents (*M* = 4.94, *SD* = 2.93; Z = -2.350, *p* = 0.019) at baseline. No other differences were found in baseline measures and the number of MB sessions attended between ‘at-risk’ and not ‘at-risk’ parents.

#### Differences between mothers and fathers

Comparisons between mothers and fathers (both in completers’ and non-completers’ analyses) found no significant differences in baseline scores and attendance, except for the ‘Tunnel’ scores in the completers’ analysis. Specifically, father’s perception of closeness with their babies at baseline (*M* = 4.31, *SD* = 2.81) was significantly lower than that of mothers (*M* = 6.33, *SD* = 2.58; *U* = 96.50, *p* = 0.034). However, after using MI to impute the missing values in the dataset, baseline ‘Tunnel’ scores for mothers (*M* = 6.50, *SD* = 2.41) and fathers (*M* = 5.12, *SD* = 3.21) showed no significant gender difference (*U* = 491.00, *p* = 0.065).

Further comparisons between completers and non-completers indicated that non-completers had significantly higher baseline KPCS scores (*t*(89) = -2.65, *p* = 0.01).

### Psychological and parenting outcomes

No significant differences were found between mothers’ and fathers’ pre-post-intervention main outcomes; therefore, data were combined. Findings from our completer and intention-to-treat analyses can be found in Table 2.

**Table 2:** Mean pre-and post-group scores for all outcome measures for the completer sample and imputed dataset.

Outcome Measure	N	Pre mean (SD)	Post mean (SD)	*t*	*Z*	*d*
**BSI-18 Total**	complete, *n* = 69	56.30 (10.76)	53.71 (11.58)	2.591*		0.312
	imputed, *n* = 91	56.70 (10.40)	53.99 (10.12)		-2.823*	0.313
Depression	complete, *n* = 69	55.39 (11.09)	53.16 (11.03)		-1.868	
	imputed, *n* = 91	56.03 (10.73)	53.55 (9.64)		2.823*	0.261
Anxiety	complete, *n* = 69	56.65 (11.25)	53.25 (12.48)	2.473*		0.298
	imputed, *n* = 91	56.65 (10.67)	53.66 (10.93)		-2.303*	0.273
Somatization	complete, *n* = 69	52.30 (10.91)	51.51 (10.47)		-0.701	
	imputed, *n* = 91	52.80 (10.95)	51.62 (9.17)		-1.062	
**Q-LES-Q-SF**	complete, *n* = 69	48.81 (7.87)	48.94 (9.25)	-0.155		
	imputed, *n* = 91	47.87 (8.74)	48.86 (8.08)		-0.674	
**KPCS**	complete, *n* = 69	38.81 (3.51)	40.30 (3.06)	-3.776*		0.455
	imputed, *n* = 91	39.29 (3.49)	39.78 (2.84)	-1.288		
**‘Tunnel’**	complete, *n* = 37	5.51 (2.82)	8.61 (1.15)			
	imputed, *n* = 85	6.16 (2.68)	8.86 (0.98)		-7.084*	1.337
Mothers	complete, *n* = 22	6.33 (2.58)	8.69 (1.14)		-3.376*	1.183
	imputed, *n* = 64	6.50 (2.41)	8.95 (0.92)			
Fathers	complete, *n* = 15	4.31 (2.81)	8.49 (1.19)		-3.352*	1.940
	imputed, *n* = 21	5.12 (3.21)	8.57 (1.10)			

BSI-18: Brief Symptom Inventory [36]; KPCS: Karitane Parenting Confidence Scale [40]; Q-LES-Q-SF: Quality of Life Enjoyment and Satisfaction Questionnaire–Short form [42, 43]; complete = complete case; imputed = imputed values; d = within-group Cohen’s d effect sizes; SD = Standard Deviation; t = paired-samples t-test statistic

*p < 0.05; Z = Wilcoxon signed-rank test.

#### ‘Completer’ analysis

Paired-sample t-tests (using complete case data only, *n = 69*), yielded significant pre-post intervention improvements on BSI-18 Global Severity Index (*t*(68) = 2.59, *p* = 0.012, *d* = 0.31) and KPCS (*t*(68) = -3.776, *p* < 0.001, *d* = 0.46) scores, with medium effect sizes. A chi-square test also showed that the distribution of parents in the four KPCS categories (i.e. ‘non-clinical’, ‘mildly clinical’, ‘moderately clinical’ and ‘severely clinical’) was significantly different, with fewer cases in the clinical categories at T2 compared to baseline (*χ*^*2*^(3) = 9.11, *p* = 0.028). From the three BSI-18 subscales, only the Anxiety subscale showed a significant reduction over time (*t*(68) = 2.47, *p* = 0.016, *d* = 0.30). As baseline Depression and Somatization subscale scores were non-normal (Wilks-Shapiro test *p* < 0.001), we also performed a Wilcoxon signed-rank test, which confirmed a non-significant pre-post increase in confidence (*Z* = -1.87, *p* = .054, *p* = 0.054 and *Z* = -0.70, *p* = 0.434 respectively). There was no significant change in Q-LES-Q-SF scores over time (*t(*68) = -0.155, *p* = 0.877).

#### Intention-to-treat (ITT)–Multiple imputation (MI)

To account for the parent-infant dyads who dropped out from either the program or the study, multiple imputation was used to estimate the post-outcome scores missing in the data.

Results from the BSI-18 completer analysis remained similar when using MI for the Global Severity Index *t*(90) = 2.99, *p* = 0.04, *d* = 0.31) and the Anxiety subscale (*t*(90) = 2.60, *p* < 0.011, *d* = 0.27), but not for the Depression subscale, which was statistically significant using MI (*t*(90) = 2.49, *p* = 0.015, *d* = 0.26) compared to a non-significant trend in the completer analysis (*p* = 0.053). The BSI-18 Somatization subscale (*t*(90) = 1.24, *p* = 0.219) and Q-LES-Q-SF (*t*(90) = -1.15, *p* = 0.254) did not exhibit significant changes over time. Wilcoxon signed-rank tests were also carried out for the baseline measures which violated normality. These confirmed a significant reduction over time in the levels of Global Severity Index (*Z* = -2.823, *p* = 0.005), Anxiety subscale (*Z* = -2.30, *p* = 0.021), and Depression subscale (*Z* = -2.28, *p* = 0.023), and a non-significant one for the Somatization subscale (*Z* = -1.06, *p* = 0.288) and Q-LES-Q-SF (*Z* = -0.67, *p* = 0.500).

Contrary to the completer analysis, the MI analysis found no significant changes in KPCS (*t*(90) = -1.29, *p* = 0.201). Wilcoxon signed-rank tests also did not reach the level of significance for differences over time (*Z* = -1.11, *p* = 0.269). Chi-square tests showed no significant differences in the distribution of parents in the clinical categories from before to after the intervention (*χ*^*2*^(3) = 4.18, *p* = 0.243).

### Socio-emotional child outcomes

#### ‘Completer’ analysis

Based on the ASQ:SE-2 baseline scores, 34 children were considered low or no risk, 15 were in the “monitor” category, 11 were recommended to be referred, and 9 were in the top 90^th^ percentile. Post-group categories distribution was shifted in the predicted way (*n* = 47, *n* = 9, *n* = 9 and *n* = 4 respectively), but it did not reach significant levels (*χ*^*2*^(3) = 5.71, *p* = 0.127).

#### Intention-to-treat (ITT)–Multiple imputation (MI)

The changes between T1 and T2 in the ASQ-SE-2 categories distribution were not significantly different over time when using MI (*χ*^*2*^(3) = 3.40, *p* = 0.334).

### Perceived parent-child relationship

#### ‘Completer’ analysis

Repeated measures analysis was carried out separately for mothers and fathers who completed the ‘Tunnel’ at all three time points (*n* = 37), as baseline scores in the completer dataset were significantly different. As this data violated assumptions of normality (*p* = 0.042), Friedman tests were used. A statistically significant difference in ‘Tunnel’ scores was found over time both for mothers (*χ*^*2*^(2) = 20.32, *p* < 0.001) and fathers (*χ*^*2*^(2) = 2.24, *p* < 0.001). Post-hoc analysis was carried out with a Bonferroni correction applied to results; as such, statistical significance level was set at 0.017. Analyses indicated that the changes in mother scores from T1 to TM were not significant (*Z* = -1.63, *p* = 0.102), whereas the changes from TM to T2 and from T1 to T2 were significant with a large effect size (*Z* = -3.90, *p* = 0.001, *d* = 1.043 and *Z* = -3.90, *p* < 0.001, *d* = 1.18 respectively). Similarly, post-hoc tests for father scores showed a non-significant change from T1 to TM (*Z* = -1.85, *p* = 0.064), but a significant change from TM to T2 (*Z* = -3.41, *p* = 0.001; *d* = 1.75) and from T1 to T2 (*Z* = -3.35, *p* = 0.001, *d* = 1.94). There was no significant difference between the scores reported by mothers and fathers at T2 (*Z* = -0.78, *p* > .017). Also, differences at T2 were no longer significant between ‘at-risk’ (*M* = 8.94, *SD* = 1.08) and not ‘at-risk’ (*M* = 8.90, *SD* = 1.04) parents (*Z* = -0.086, *p* = 0.931).

#### Intention-to-treat (ITT)–Multiple imputation (MI)

Data from mothers and fathers were combined for the ITT analysis as there were no baseline gender differences (*n* = 85). Assumptions of normality were again violated (*p* = 0.004); therefore, Friedman tests were carried out indicating a significant difference between T1, TM, and T2 reported rates (*χ*^*2*^(2) = 80.56, *p* < 0.001). Post-hoc tests identified that changes between each time point were statistically significant with a large effect size (T1 to TM (*Z* = -4.11, *p* < 0.001, *d* = 0.49), TM to T2 (*Z* = -7.54, *p* < 0.001, *d* = 1.09) and T1 to T2 (*Z* = -7.084, *p* < 0.001, *d* = 1.34).

### Group satisfaction and involvement with child protection services

Post-intervention data on participants’ subjective experience of MB was available for all 69 parents who completed the intervention (see S1 Table). Of those, 98.5% (68) agreed or strongly agreed with the statement “I enjoyed taking part in the Mellow group”, 87% (60) with the statement “I feel more connected with my child after taking part in this group”, and 92.7% (64) with the statement “I feel confident in asking for help should I need it”.

Changes in the involvement with child protection services throughout the program were available for 76 participants. Data from facilitators indicated that 65.8% (50) of the parents were not involved in child protection services over the course of the program. Of those involved (26), 57.7% (15) experienced a de-escalation of their case, 3.8% (1) underwent a step up in their child protection involvement, and 38.5% (10) had no change to the level of social care usage from the beginning to the end of the program. A chi-square test demonstrated that this distribution was significantly different from the expected distribution if all categories were equal (*χ*^*2*^(2) = 11.62, *p* < 0.05).

## Discussion

This is the first study to prospectively evaluate the impact of Mellow Babies in a ‘real-life’ context across fifteen Mellow Mums and Mellow Dads groups delivered in the UK. Outcomes suggest that MB can be effective for both mothers and fathers, including families with higher levels of risk factors. Completion of MB was associated with significantly reduced parental psychological distress and anxiety levels, along with increased parenting confidence and perceived closeness of the parent-infant relationship. In addition to these, ITT analyses indicated significant improvements in parental depression symptoms post-intervention, although parenting confidence changes no longer reached statistical significance. Attendance and intervention satisfaction was high among completers, indicating that, from an implementation science perspective, MB is accessible and acceptable to parent-baby dyads with complex needs.

Evidence consistently highlights the interlinking and multilevel barriers to accessing and engaging new parents with complex needs in postnatal services [49, 50]. Nevertheless, we note that our attrition rates were generally lower than those reported for parenting programs delivered in the UK and internationally [51–53], despite the majority of our sample considered to be ‘at-risk’ based on the psychosocial criteria set. Additionally, contrary to the existing body of research suggesting that fathers are a challenge to recruit and engage in parenting programs [15–17] this study reported particularly favorable father recruitment and involvement (1/3 of total MB groups). Once engaged in the program, fathers were also less likely to drop out compared to mothers (9.1% vs 28.6%). This is substantially lower than estimates of 26% dropout rates reported in a recent meta-analysis [51] and mirrors findings reporting no gender dropout differences in parenting programs [52].

MP programs target the referral of parents at high risk of adversity and employ a range of strategies to encourage their participation and engagement. One of the strengths of MB that differentiates it from similar early intervention parenting programs is the provision of transport, childcare, low-literacy materials, and daily meals to all parent-baby dyads enrolled in the groups which can positively contribute to overcoming attendance and commitment barriers, usually encountered by ‘at-risk’ parents [49]. Furthermore, as identified in previous studies, participating in a group of parents with similar experiences that is facilitated in a non-judgmental, tailored, and accessible way—as MB is—allows parents to actively engage with the program and be open to changes [52–55]. Our study demonstrates that, regardless of gender and ‘at-risk’ status, all parents can benefit equally from attending a program that is specifically designed to engage them, in contrast to studies suggesting that the effects of attending a parenting program may be more positive for mothers [53].

Our findings are consistent with the emergent worldwide focus on perinatal (i.e. the period encompassing pregnancy through to the end of the first postpartum year) mental health and support recommendations that parenting programs should strive to improve parental mental health as well as promoting sensitivity in parent-child relationships through interaction guidance [56, 57]. Although we did not explore the specific MB mechanisms of change, previous evidence indicates that components such as including a support group, using individualized video-feedback, and incorporating more than one intervention component can predict the effectiveness of interventions targeted at mothers experiencing mental health difficulties and difficulties in the relationship with their babies [58, 59].

The present study advocates that the same intervention components can also be effective for improving fathers’ wellbeing and confidence in their parenting role, building upon the medium-level effect sizes of MP on maternal mental health and parenting confidence [29–32]. While traditionally mothers’ mental health has received greatest attention, recognition of the importance of fathers’ mental health and its role to offspring’s development is gathering momentum [19, 60], acknowledging that fathers can affect their children both directly, via their genes and quality of their interactions, and indirectly, via their support to the mother and family environment [5, 10]. It is therefore of high importance to continue providing opportunities for involving fathers in parenting programs and in parenting more generally.

Examining the effect of MB with the imputation of missing values for the parents who did not complete the intervention further validated our findings. Specifically, our results remained consistent across ITT analyses, except for the improvement in parenting confidence. This raises questions around potential reasons for participant dropout, given that non-completers reported a higher level of parenting confidence before starting MB, altering our findings when included in the analyses. These, however, are beyond the scope of this study.

Improvements in self-competence among MB completers is considered a key parenting outcome, given that parenting confidence is identified as an important intrapersonal resource associated with better parent psychological health [61], quality of parenting behavior [62], and overall baby developmental outcomes [63]. The specific direction of change, nevertheless, remains unclear. Longitudinal assessments support child-to-parent (rather than parent-to-child) effects, indicating that mothers' confidence in their ability to parent is mainly influenced by their experience with a difficult infant and by their depressive symptoms during the child's first year of life [64]. In turn, depressive symptoms can be aggravated by mothers' low perceived parenting confidence [64]. Further understanding of the bidirectional nature of these constructs could provide a more accurate interpretation of intervention effects and enable services to develop more effective and targeted early intervention strategies for parents and their babies.

We also note that parents in the study reported increased perceived connectedness with their child, moving closer towards their ‘ideal’ relationship over the course of MB. This was reflected both in the “Tunnel” task completed by the parents at three time points and in the post-intervention feedback. Also, over half of the parents involved with child protection services witnessed a de-escalation of their case by the end of the group. This ‘real-life’ indicator suggests that statutory sector services, primarily the ones who also referred parents to MB groups, identified signs of improvement in participants’ parenting skills and relationship quality with their children, leading to decisions of decreasing their involvement with child protection services. The contribution of MP group facilitators in the re-evaluation of the participants’ cases is usually central, given their capacity to provide evidence for the parent-child interaction quality and parents’ behavioral and emotional progress over the preceding three months. Therefore, this observation may also act as a proxy indicator of MB effectiveness. ‘At-risk’ parents and, specifically, parents experiencing deterioration of their mental health during the first postnatal months, can find it difficult to focus on their infant, notice their signals and interests, and respond appropriately [65, 66]. Providing support to parents to reduce the levels of their symptoms during the MB intervention can, therefore, also allow them to tune-in better to their babies’ emotional and practical needs and positively impact the way they perceive the quality of their relationship [10]. Given the use of non-standardized measures, we urge some caution in interpreting improvements of parent-child relationship quality for MB participants, however, we expect that the qualitative data and video observation recordings collected during this project will elucidate this outcome.

Additionally, our findings indicate no significant immediate post-intervention changes in children’s socio-emotional development, highlighting the need for longitudinal designs when exploring child outcomes. This is consistent with evidence that, in contrast to parenting sensitivity and parent-child relationships, which can be improved within a relatively short time [67], the effects of parenting interventions on child development may take longer to emerge. Despite the wide variation in intensity and duration of early interventions directed primarily at parents, a meta-analysis shows that effects on children’s internalizing symptoms can be maintained for up to 11 years post-intervention [68]. Therefore, further longitudinal research on the impact of MB on children’s developmental outcomes is required.

Similarly, there was no significant effect on the overall quality of life, enjoyment, and satisfaction post-intervention, contrary to findings from other parenting programs [69]. The holistic nature of the outcome measure used in this study, including aspects of life that are not directly targeted at MB, (such as housing conditions, and ability to get around without feeling dizzy or unsteady), may have resulted in overall changes taking longer to emerge. The feedback questionnaire indicates that the vast majority of participants intended to remain engaged with parenting services and reported feeling more confident to seek professional help should they need it. This suggests that MB participants developed trusting relationships with group practitioners and services, reducing access barriers usually associated with help-seeking during the perinatal period [49, 50]. We note that MB promotes the continued involvement of parents with services, and group facilitators provide tailored support to parents upon program completion, directing them to relevant self-development and family-focused initiatives if deemed appropriate. Qualitative and follow-up data would be more appropriate to shed light on the effects of MB on the overall quality of parent’s lives.

### Limitations and future directions

Evaluating the effectiveness of an early parent-infant intervention targeted at both mothers and fathers experiencing psychosocial difficulties is in line with the growing international public health interest in preventive strategies and the increasing perinatal focus of national policy frameworks across both high and low resource settings [70–72]. However, this study also has several limitations. Data were collected in a ‘real world’ setting, therefore lacking a control group for comparison. Consequently, we cannot assume that findings were directly caused by the MB intervention. Additionally, the number of mothers and fathers included in this study were not equal. However, this mirrors the general tendency for parenting groups to be mainly offered to mothers. Future studies should aim to replicate our results with the inclusion of a control or contrast group and, if possible, an equal gender distribution. Furthermore, this study only assessed the intervention effects immediately after MB completion, making it impossible to reach conclusions for the long-term effects of MB and possibly underestimating its effects in areas that need more time to emerge, such as child developmental outcomes. Future intervention evaluations should include follow-up data to assess the lasting benefits of the program, utilizing direct objective measures to assess parent-child relationships and interactions (e.g. interaction videos). Due to the low internal reliability found in the parenting confidence scale and two of the five developmentally-specific child socio-emotional questionnaires in our sample, our findings for these outcomes should also be treated with caution and require replication. The low internal reliabilities on these specific scales could be due to a low number of scale items, poor inter-relatedness between items, or because items within the scale or subscales may not measure the putative underlying constructs. Finally, future research should assess the cost-effectiveness of MB and consider the implementation of wholly or hybrid digital formats for the intervention. Although a relatively resource-intensive program, MB shows the potential to help reduce symptom severity of perinatal mental health difficulties and involvement with child protection services, suggesting long-term savings that may offset the initial cost of implementing the program [73].

## Supporting information

S1 Table Feedback questionnaire results. (DOCX)

S1 Dataset Mellow Babies project data. (XLSX)
