# Supplementary material for: Promoting sensitive parenting in ‘at-risk’ mothers and fathers: A UK outcome study of Mellow Babies, a group-based early intervention program for parents and their babies
Source: PLoS One. 2021 Feb 3;16(2):e0245226. doi: 10.1371/journal.pone.0245226 (PMC7857589; doi:10.1371/journal.pone.0245226)
Supplement: S1 Table — (DOCX) [file pone.0245226.s001.docx]

**S1 Table. Feedback questionnaire results.**

| **Question n (%)** | **Strongly Disagree** | **Disagree** | **Neither agree nor disagree** | **Agree** | **Strongly Agree** |
| --- | --- | --- | --- | --- | --- |
| 1. I enjoyed taking part in the group. | 0 (0%) | 0 (0%) | 1 (1.5%) | 9 (13.2%) | 59 (85.3%) |
| 2. I found the morning sessions helpful.^1^ | 0 (0%) | 1 (1.4%) | 2 (2.9%) | 13 (18.8%) | 53 (76.8%) |
| 3. I found the afternoon sessions helpful.^2^ | 0 (0%) | 1 (1.5%) | 4 (5.9%) | 12 (17.6%) | 51 (75.0%) |
| 4. I found the lunchtime activities helpful. | 0 (0%) | 2 (2.9%) | 4 (5.9%) | 15 (22.1%) | 47 (69.1%) |
| 5. I would like to keep in touch with the service who delivered this group. | 1 (1.4%) | 0 (0%) | 0 (0%) | 14 (20.3%) | 54 (78.3%) |
| 6. I would like to keep in touch with the other group members. | 0 (0%) | 2 (2.9%) | 5 (7.2%) | 7 (10.1%) | 55 (79.7%) |
| 7. I feel more connected with my child after taking part in this group. | 1 (1.4%) | 0 (0%) | 8 (11.6%) | 12 (17.4%) | 48 (69.6%) |
| 8. I feel confident in asking for help should I need it. | 0 (0%) | 0 (0%) | 5 (7.2%) | 15 (21.7%) | 49 (71.0%) |
| 9. I feel the children’s group has been beneficial for my child. | 0 (0%) | 0 (0%) | 1 (1.5%) | 9 (13.2%) | 58 (85.3%) |
| 10. I found the experience of sharing my video with other parents helpful. | 1 (1.7%) | 2 (3.4%) | 5 (8.5%) | 15 (25.4%) | 36 (61.0%) |

^1^Parent focused session; ^2^Parenting focused session (i.e. strength-based video feedback)
